# External Focus Affects Drop Jump Performance: Focusing on Different Aims and Words of Instruction

**DOI:** 10.5114/jhk/159235

**Published:** 2023-07-06

**Authors:** Yuki Furuhashi, Yusuke Hioki, Hirohiko Maemura, Ryohei Hayashi

**Affiliations:** 1 Graduate School of Comprehensive Human Sciences, University of Tsukuba, Ibaraki, Japan.; 2 Joint Graduate School in Science of School Education, Hyogo University of Teacher Education, Hyogo, Japan.; 3 Faculty of Health and Sports Science, University of Tsukuba, Ibaraki, Japan.; 4 Faculty of Education, Gifu University, Gifu, Japan.

**Keywords:** biomechanics, stretch-shortening cycle, plyometric training, kinetics, attentional focus strategies

## Abstract

Attentional focus strategies eliciting external focus of attention effectively enhance drop jump (DJ) performance, however, their effects vary depending on the words used for the instructions. We aimed to examine the effects of different words on DJ performance using instructions eliciting external focus to minimize contact time (CT) or maximize jump height (JH). Twenty collegiate athletes performed DJs from a 30-cm platform after receiving one of the four instructions: two instructions (COND 1 and 2) about minimizing CT, and two instructions (COND 3 and 4) about maximizing JH. The reactive strength index (RSI), CT, JH, relative peak vertical ground reaction force (vGRF), and leg stiffness (k_vert_) were compared between conditions using repeated-measures analysis of variance. There was no significant main effect of conditions on the RSI, relative peak vGRF, and k_vert_ (p > 0.05). CT was significantly shorter under CONDs 1 and 2 than COND 3 (p < 0.05); JH was significantly higher under COND 3 than CONDs 1 and 2 (p < 0.05), and under COND 4 than COND 1 (p < 0.05). When using attentional focus strategies in DJ, it is necessary to use different words and purposes according to the players' tasks.

## Introduction

Plyometric training is a common and effective method for improving jumping ability by enhancing stretch-shortening cycle (SSC) mechanics (Ciocca et al., 2021; Makaruk et al., 2012). A drop jump (DJ) is a plyometric exercise that involves stepping from a predetermined height, landing, and immediately performing a maximum vertical jump (Ball et al., 2010). The DJ is used by strength and conditioning (S&C) coaches as a training method to assess an athlete's SSC ability (Flanagan and Comyns, 2008). A high-performance DJ requires minimum contact time (CT) upon landing and maximum jump height (JH) immediately after that (Flanagan and Comyns, 2008). Analysis of DJ performance includes using the reactive strength index (RSI), calculated by dividing JH by CT (Young, 1995). A previous study found that the RSI had a strong correlation with the 100-m sprint time and change-of-direction speed (Hennessy and Kilty, 2001; Young et al., 2015). DJ training improves lower extremity maximal muscle strength, vertical jumping ability, and short sprinting ability (Chelly et al., 2010). Therefore, the DJ is an important training approach to enhance performance in many sports.

Leg stiffness (k_vert_) is related to a fast SSC (Comyns et al., 2007); k_vert_ is defined as the ratio of the peak vertical ground reaction force (vGRF) to the maximum vertical displacement of the center of mass during foot contact with the ground (Kerdok et al., 2002), and its optimization is an important technical aspect of DJ performance (Ferris and Farley, 1997). A correlation between k_vert_ and the RSI in DJ has been previously reported (Kipp et al., 2010). Thus, k_vert_ can be a useful and reliable indicator for assessing DJ performance.

Attentional focus strategies can change one's focus of attention during exercise and change athletic performance (Wulf, 2013). Verbal instructions can promote either neutral, internal or external focus of attention. Internal focus of attention refers to directing one's attentional focus to specific body parts and their movements; external focus of attention refers to directing one's attention to the effects of the exercise environment; and neutral focus of attention does not aim to induce either internal or external focus, but instead aims to promote nonawareness (Wulf, 2008). Numerous studies on attentional focus strategies in various sports have shown that using external focus of attention rather than internal focus of attention improves performance (Wulf, 2008, 2013; Wulf and Dufek, 2009). These results can be explained by the constrained action hypothesis (McNevin et al., 2003), which suggests that when the internal focus of attention is used, the motor control system is constrained, thus interfering with the automatic motor processes that control movement (Porter et al., 2015). Therefore, choosing an attentional focus strategy is important for high performance in many sports.

An attentional focus strategy is also effective for DJ performance. Previous studies reported that the RSI, maximum ground reaction force, CT, and k_vert_ were improved by external focus of attention (Comyns et al., 2019; Furuhashi et al., 2022). Oliver et al. (2021) demonstrated that during the DJ, instructions on minimizing CT (which elicit external focus of attention) led to shorter CT than other conditions, and instructions on maximizing JH induced greater JH than other conditions (Oliver et al., 2021). Thus, verbal instructions eliciting external focus of attention can improve the RSI of the DJ, as well as CT and JH. However, it has been reported that when instructions are too complex, the attentional focus strategy is not fully effective (Wulf, 2007). Indeed, careful word choice is important because appropriate instructions can “load working memory” and promote proper processing and attention to the skill (Furley and Wood, 2015). Therefore, to improve performance of each individual, S&C coaches need to select appropriate instructions. Various instructional content attentional focus strategies are also used in actual training fields, both for the internal and external focus of attention (Porter et al., 2010). However, no studies have examined multiple verbal instructions to determine which ones are more effective in improving performance.

Based on previous reports on attentional focus strategies and instructions (Comyns et al., 2019; Furuhashi et al., 2022; Gawel et al., 2022; Oliver et al., 2021; Siembida et al., 2021), we chose to focus on the external focus of attention, which is associated with high DJ performance, to clarify the effects of different contents of instructions on DJ performance. We hypothesized that verbal instructions that elicit external focus of attention (e.g., minimize CT, maximize JH) would have different effects on DJ performance depending on the words of the instructions, even if the aim was the same. Given the report of Oliver et al. (2021), CT may decrease when instructions to shorten it are given, and JH may increase when such instructions are provided. We aimed to examine the effects of different words on DJ performance using multiple verbal instructions eliciting the external focus of attention to minimize CT or maximize JH.

## Methods

### Participants

Twenty male collegiate-level athletes (six soccer players, six handball players, four swimmers, three kendo players, and one basketball player) participated in this study. The exclusion criteria were as follows: use of medication affecting exercise capacity, and/or orthopedic limitations. Table 1 shows the participants’ physical characteristics. Their training was sport-specific and occurred over five days a week. Participants were familiar with the DJ technique and had intermediary experience with this exercise. Informed consent was obtained from all participants. The consent form and all experimental methods were approved by the Research Ethics Committee of the University of Tsukuba prior to the initiation of this study (tai 019-18).

**Table 1:** Physical characteristics of participants (mean ± *SD*).

Age (y)	Body height (m)	Body mass (kg)
21.8 ± 1.5 (19–24 years old)	1.73 ± 0.05	69.7 ± 5.6

### Design and Procedures

The experiment was performed on two separate days. On the first day, two conditions were measured. On the second day, which was 3–5 days after the first day, the other two conditions were measured. All participants were instructed to refrain from resistance training the day before the experiment. Before the experiment, participants performed 15 minutes of low-intensity jogging and dynamic stretching, followed by five DJs using a 30-cm box (Khuu et al., 2015). Participants were instructed to stand on a 30-cm box, and the examiner read the DJ instructions for external focus of attention aloud. The verbal instructions were drafted based on previous studies (Comyns et al., 2019; Oliver et al., 2021), and are listed in Table 2. For conditions 1 (COND 1) and 2 (COND 2), the instructions were created to minimize CT, and for conditions 3 (COND 3) and 4 (COND 4), the instructions were created to maximize JH. Verbal instructions for each condition were always reread before each trial. On the platform, the posture of the preset phase was standardized for each trial by marking each participant’s eye height on the wall. All participants uniformly performed the test barefoot. Participants were instructed to keep their hands on their hips, bend their knees as little as possible, and perform every DJ with maximal intensity (Bergmann et al., 2013). Participants performed two DJs under each condition, with a total of eight DJs across the four conditions. If the participant explicitly failed the DJ, such as if the participant landed off the force plate, the trial was repeated. Participants were given a 1-min rest interval between trials under the same conditions, and a 2-min rest interval between each condition (Amasay and Suprak, 2022; Comyns et al., 2019). Participants were not provided with any feedback regarding their performance.

**Table 2:** Drop jump verbal instructions designed to invoke external focus of attention.

Conditions	Verbal instructions
**COND 1**	“Focus on spending as little amount of time on the floor as possible.”
**COND 2**	“Imagine the ground is a hot surface, get off the ground as quickly as possible.”
**COND 3**	“Focus on getting as close to the ceiling as possible.”
**COND 4**	“Imagine you are like a stiff spring and focus on jumping to the roof.”

COND = Conditions

### Measures

JH, CT, RSI, and k_vert_ values were used to evaluate DJ performance. The vGRF values were collected using a force plate (PH-6210A, 0.9 m × 0.9 m: DKH, Tokyo, Japan) measuring at 1,000 Hz. Values for flight time, CT, peak vGRF, and the center of mass were obtained directly from the force plate data. The peak vGRF was divided by the participant’s mass to derive the relative peak vGRF. JH was defined as the flight time component, and was determined using the equation: JH = (9.81 × flight time^2^) / 8 (Flanagan and Comyns, 2008). The RSI for each DJ trial was calculated by dividing JH in meters by CT in seconds (Butler et al., 2003). The mass-spring model was used to calculate k_vert_, according to the method described by Comyns et al. (2011), where the peak vGRF was divided by the displacement of the participant from the initial downward movement to the lowest point of the center of mass during recovery. The average of the two trials was used for further analysis.

### Statistical Analysis

All statistical analyses were conducted using IBM SPSS Version 25 (IBM, New York, NY, USA). Reliability of performance measures was assessed by the intraclass correlation coefficient (ICC) using 90% confidence intervals (90% CI). An ICC over 0.90 was considered high, between 0.80 and 0.90 moderate, and below 0.80 insufficient for physiological field testing (Vincent, 2020). Data were normally distributed for all conditions, as assessed by the Shapiro-Wilk test (*p* > 0.05). One-way analysis of variance (ANOVA) was used to examine the data for inter-condition differences. If the assumption of sphericity was rejected using the Mauchly's test of sphericity, the Greenhouse-Geisser correction was applied. When a statistically significant main effect was detected, Bonferroni's post hoc tests were performed using pairwise comparisons of alpha levels to identify differences in variable scores between conditions. Effect sizes using Hedges' g were obtained for any significant pairwise comparisons and interpreted using the following scale: less than 0.2, trivial; between 0.2 and 0.5, small; between 0.5 and 0.8, medium; between 0.8 and 1.3, large; and greater than 1.3, very large (Cohen, 1988). All data are presented as mean ± standard deviation (*SD*).

## Results

The ICC and 90% CI between the first and second measurements of the RSI are shown in Table 3. The values of the ICC between the two trials for the RSI were high for all conditions (COND 1 = 0.928, COND 2 = 0.934, COND 3 = 0.886, COND 4 = 0.919).

**Table 3:** Intraclass correlation coefficient (ICC) and 90% confidence interval (CI) for the reactive strength index (RSI) under each condition.

	COND 1	COND 2	COND 3	COND 4
**ICC**	0.928^*^	0.934^*^	0.886^*^	0.919^*^
**90% CI**	1.60–1.93	1.61–193	1.59–1.92	1.57–1.92

COND = Conditions

* p < 0.05

Mean ± *SD* scores for all variables under the four conditions are detailed in Table 4.

**Table 4:** Mean ± standard deviation (*SD*) scores for each drop jump variable under each condition.

	COND 1	COND 2	COND 3	COND 4
**RSI (m/s)**	1.76 ± 0.43	1.77 ± 0.41	1.76 ± 0.42	1.74 ± 0.46
**CT (s)**	0.181 ± 0.029†	0.179 ± 0.026†	0.193 ± 0.0.32	0.193 ± 0.038
**JH (m)**	0.313 ± 0.064	0.312 ± 0.067	0.332 ± 0.066‡§	0.326 ± 0.066‡
**Relative peak vGRF (N/kg)**	61.5 ± 9.0	59.6 ± 8.7	58.5 ± 9.5	58.5 ± 9.5
**k_vert_ (kN/m)**	23.9 ± 9.68	23.2 ± 7.86	21.9 ± 9.42	23.7 ± 11.6

COND = Conditions; RSI = reactive strength index; CT = contact time; JH = jump height; vGRF = vertical ground reaction force; k_vert_ = leg-spring stiffness.

† significantly shorter than COND 3, p < 0.05, ‡ significantly higher than COND 1, p < 0.05, § significantly higher than COND 2, p < 0.05

Repeated measures ANOVA showed no significant main effect of conditions on the RSI (*F* = 0.13, *p* = 0.94, partial *η^2^* = 0.01), relative peak vGRF (*F* = 2.14, *p* = 0.11, partial *η^2^* = 0.10), and k_vert_ (*F* = 1.13, *p* = 0.35, partial *η^2^* = 0.06); however, there was a significant main effect of conditions on CT (*F*=6.88, *p* = 0.01, partial *η^2^* = 0.27) and JH (*F* = 8.00 *p* = 0.01, partial *η^2^* = 0.27). Post hoc pairwise comparisons found that CT was significantly shorter under COND 1 than COND 3, as well as under COND 2 than COND 3 (Table 5). JH was significantly higher under COND 3 than CONDs 1 and 2, as well as under COND 4 than COND 1.

**Table 5:** Effect size between each condition of the drop jump variable.

Variables	CT	JH
Conditions	Effect size (*p*-value)	
**COND 1 vs. 2**	n.s.	n.s.
**COND 1 vs. 3**	0.37 (0.02)	0.29 (0.04)
**COND 1 vs. 4**	n.s.	0.21 (0.01)
**COND 2 vs. 3**	0.47 (0.02)	0.29 (0.01)
**COND 2 vs. 4**	n.s.	n.s.
**COND 3 vs. 4**	n.s.	n.s.

COND = Conditions; vs. = versus;

CT = contact time; JH = jump height;

n.s. = not significant

## Discussion

We aimed to examine the effects of different words on DJ performance using multiple verbal instructions eliciting external focus of attention to minimize CT or maximize JH. Our main finding was that attentional focus strategies allowed for minimizing CT or maximizing JH without changing the RSI, relative peak vGRF, and k_vert_. The results are consistent with the hypothesis that different words used in attentional focus strategies have different effects on DJ performance, even when the aims of the strategies are the same.

Our findings suggest that all the attentional focus strategies used here might not affect the RSI during the DJ, although their aims and the words used were different. Hence, the results differed from those obtained by Oliver et al. (2021), in which the RSI was higher under the instruction of minimization CT condition, maximization JH condition, and minimization motion sound condition in that order. Furthermore, the mean values of the RSI here (1.74–1.77) and in the previous study (0.40–0.67) differed significantly (Oliver et al., 2021). The difference in results between the current and previous studies may be related to differences in the subject's specialty and age (Oliver et al., 2021). Our study participants specialized in various sports, whereas the previous study used only soccer players (Oliver et al., 2021). The participants’ DJ performance and force exertion characteristics differed depending on the participant’s specialized sports (Kollias et al., 2004). The mean age also differed significantly between the current study (21.8 ± 1.5) and the previous one (11.6 ± 0.5) (Oliver et al., 2021). Lloyd et al. (2016) reported that the process of relearning happens in athletes after a period of growth. Given these considerations, it is possible that the current study and previous ones produced different results for DJ performance and attentional focus strategies depending on participants’ characteristics.

Under CONDs 1 and 2, we utilized attentional focus strategies to minimize CT, and both conditions achieved this aim; CONDs 3 and 4 aimed to maximize JH and both conditions achieved this aim. These results partially support previous studies that reported changes in DJ performance by changing the aim of the instructions (Furley and Wood, 2015; Makaruk et al., 2012). It is important to choose words carefully as appropriate instructions can “load the working memory” and encourage processing and attention appropriate to the skill (Furley and Wood, 2015). Thus, the instructions in CONDs 1, 2, 3, and 4 were appropriately worded, which may have resulted in changes in JH and CT. Although the effect sizes (ES) were small, the values varied (ES = 0.21–0.47). This means that the degree of effect on DJ performance varied depending on the content of instructions. Thus, these results suggested the need to carefully choose words for the instructions. However, further research is needed on the details of what verbal instructions are most easily understood by athletes.

Relative peak vGRF did not differ significantly between conditions. This result differs from that reported, where impact force was higher when instructions were given regarding CT than when instructions were given regarding JH (Khuu et al., 2015; Oliver et al., 2021). Oliver et al. (2021) reported that increased peak force from verbal instructions was associated with an increased risk of injury. Considering this, the results of the current study are very interesting in that the attentional focus strategy was able to change CT and JH without increasing the risk of injury.

In addition, landing movements with high k_vert_ place a greater load on skeletal structures, and therefore, focusing on maximizing k_vert_ may increase the injury risk for athletes without muscle strength sufficient to withstand a certain level of eccentric contraction (Butler et al., 2003; Pedley et al., 2017). However, k_vert_ did not show any significant difference between conditions in this study. A more compliant musculotendinous system has a greater capacity to elongate enabling forces to be absorbed over a greater distance and time, thereby creating a cushioning effect (Roberts and Konow, 2013). Therefore, a change in k_vert_ may affect the behavior and prolong CT. But in this study, although CT changed, there was no significant difference in k_vert_. This suggests that the attentional focus strategy employed a stiffness strategy, such that CT could be shortened without changing k_vert_. Thus, it is considered that none of the four instructions used in this study increased the participant’s injury risk.

This study has several limitations. First, we used kinetic data and did not look at kinematic data. Moran and Wallace (2007) observed that the knee joint range of motion affected JH in the DJ, suggesting that movement is an important factor in enhancing DJ performance. Therefore, to introduce attentional focus strategies in plyometric training, it is necessary to clarify how attentional focus strategies affect the movements and change the RSI. Second, the current study included subjects specializing in different sports. Kollias et al. (2004) found that DJ performance was different among athletes of various sports. Therefore, various subjects may respond diversely to the use of attentional focus strategies due to differences in their daily training. In future studies, it is necessary to unify the participant's specialized sports and examine in more detail the effects of attentional focus strategies on DJ performance in athletes of each sport. Third, since only instructions that elicited external focus of attention were used here, it was only possible to examine the effect of different types of verbal instructions on DJ performance. Thus, we should have set up verbal instructions that elicited internal focus of attention and used two-way repeated measures ANOVA (awareness x type of instruction) to perform statistical processing. By doing so, for example, it would have been possible to examine both differences by awareness (internal vs. external) and the type of instruction (COND 1–4). This would have revealed more appropriate feedback from verbal instructions. However, the results show that using attentional focus strategies when performing the DJ is unlikely to affect the injury risk, which is helpful information for S&C coaches.

## Conclusions

By eliciting external focus of attention in DJs and changing the aim and word of the instructions given, it was possible to minimize CT and/or maximize JH without changing the RSI or k_vert_. This study’s results suggest that a simple intervention, such as using an attentional focus strategy, could solve an athletes' task of minimizing CT and maximizing JH without increasing the risk of injury.
